# Zuojinwan ameliorates depressive-like behavior and gastrointestinal dysfunction in mice by modulating the FXR-bile acid-gut microbiota pathway

**DOI:** 10.3389/fmicb.2025.1576799

**Published:** 2025-06-04

**Authors:** Dan Qiao, Bo Chen, Yuzhen Huang, Kunhan Su, Hao Wu, Weiwei Tao, Wanli Liu

**Affiliations:** ^1^Nanjing University of Chinese Medicine, Nanjing, China; ^2^Department of Gastroenterology, Affiliated Hospital of Integrated Traditional Chinese and Western Medicine, Nanjing University of Chinese Medicine, Nanjing, China; ^3^Department of Gastroenterology, Nanjing First Hospital, Nanjing Medical University, Nanjing, China; ^4^Department of Gastroenterology, Nanjing Integrated Traditional Chinese and Western Medicine Hospital, Nanjing, China; ^5^School of Integrated Chinese and Western Medicine, Nanjing University of Chinese Medicine, Nanjing, China

**Keywords:** CSDS, ZJW, depression, gastrointestinal dysfunction, bile acids, gut microbiota

## Abstract

Zuojinwan (ZJW), a traditional herbal formulation, is widely applied in the management of depression and gastrointestinal dysfunction. However, its underlying mechanisms remain inadequately characterized. This study investigated the antidepressant and gastrointestinal modulatory effects of ZJW via the bile acid–gut microbiota axis. A murine model exhibiting both depressive-like behavior and gastrointestinal disturbances was induced through chronic social defeat stress (CSDS). Antidepressant efficacy was evaluated using a battery of behavioral assessments, while gastrointestinal function was assessed by measuring total gastrointestinal transit time, colonic motility, gastric residual volume, and small intestine propulsion rate. Bile acid (BA) levels in the brain, gut, and serum were quantified using UPLC-MS/MS, and gut microbiota composition was analyzed via 16S rRNA sequencing. Histopathological examination and Western blotting (WB) were employed to assess tissue integrity and FXR protein expression, respectively. ZJW markedly ameliorated depressive behaviors and gastrointestinal dysfunction, concurrently mitigating intestinal and hippocampal neuronal damage. Treatment enhanced FXR expression, altered BA profiles, and enriched gut microbial diversity. Notably, co-treatment with the FXR inhibitor (Z)-Guggulsterone diminished the therapeutic effects of ZJW, underscoring the role of FXR signaling. These findings suggest that ZJW exerts its therapeutic benefits by activating FXR, modulating bile acid metabolism, and reshaping the gut microbiota.

## 1 Introduction

Depression is a debilitating psychiatric disorder characterized by persistent and profound low mood, often accompanied by somatic symptoms and, in severe cases, suicidal ideation or behavior. Globally, depressive disorders affect approximately 322 million individuals, representing 6.2% of the total disease burden (Friedrich, 2017). Standard treatment strategies largely rely on pharmacological and psychotherapeutic approaches (Monroe and Harkness, 2022). However, the adverse effects associated with antidepressant medications frequently compromise treatment adherence, underscoring the need for alternative therapies with favorable safety profiles and comparable efficacy (Cheng et al., 2023).

Bile acids (BAs), steroid-derived molecules synthesized in the liver, are increasingly recognized for their multifaceted roles in both physiological and pathological contexts (Thomas et al., 2008). Recent studies have highlighted their function as signaling mediators within the gut–brain axis (Monteiro-Cardoso et al., 2021), with dysregulation of BA metabolism emerging as a contributory factor in the pathogenesis of depression (Idahosa et al., 2024). The farnesoid X receptor (FXR), a nuclear receptor responsive to BAs, is broadly expressed across multiple tissues and serves as a central regulator of BA biosynthesis (Wang et al., 2008). Notably, diminished FXR expression in the prefrontal cortex has been observed in depressive mouse models, and its modulation significantly influences depression-related behaviors, identifying FXR as a promising therapeutic target (Bao et al., 2021). Beyond its role in metabolic regulation, FXR contributes to the bidirectional communication of the gut–brain axis, thereby affecting mood states via its impact on both central and peripheral systems (Idahosa et al., 2024). Altered BA profiles have been documented in both serum and brain tissues of depressed mice (Li X. Y. et al., 2024), and therapeutic manipulation of BA metabolism has demonstrated efficacy in mitigating depression-like symptoms (Zhou et al., 2023). Additionally, BAs modulate intestinal epithelial integrity, a key factor in gastrointestinal homeostasis and responsiveness to external stimuli, further linking BA dynamics to gut-related pathophysiology (Hegyi et al., 2018).

Bile acids are crucial in regulating the diversity and stability of the gut microbiota, thereby influencing its composition (Guo et al., 2022). Often referred to as the “second brain,” the gut microbiota significantly impacts both psychological and physiological health (Kahrstrom et al., 2016). Dysbiosis, or an imbalance in gut microbiota, has been implicated in the pathogenesis of various mental health disorders. Disruptions in gut microbiota composition can lead to increased intestinal permeability, activation of systemic inflammation and immune responses, and alterations in neurotransmitter release, all of which are associated with the development of depression and gastrointestinal dysfunction (Du Toit, 2022; Liu et al., 2021; Shen et al., 2024). Maintaining intestinal barrier integrity and microbial balance has been shown to alleviate symptoms of both depression (Zhou et al., 2020) and gastrointestinal disorders (Bai et al., 2023).

Zuojinwan (ZJW) is a well-established formulation in Traditional Chinese Medicine (TCM) with a long history of clinical application. First documented in Zhu Zhenheng’s Danxi’s Mastery of Medicine in 1,347, ZJW consists of two main ingredients: Huang Lian (*Coptis chinensis* Franch) and Wu Zhuyu (*Evodia rutaecarpa* (Juss.) Benth) (Wang et al., 2023). Initially, ZJW was not targeted for specific conditions but was recognized for its ability to clear liver fire and relieve liver stagnation. It was traditionally used to treat symptoms of liver Qi stagnation and excess liver fire, such as hypochondriac and abdominal distention, as well as dry mouth. In contemporary applications, ZJW has shown efficacy in treating a variety of conditions, including mental health disorders and gastrointestinal diseases (Chen et al., 2024; Dai et al., 2023).

Experimental research has demonstrated that ZJW exerts neuroprotective effects by enhancing hippocampal 5-HT and TPH2 expression, improving depressive behaviors in mice (Wang et al., 2023). Additionally, ZJW promotes MyD88 ubiquitination, suppressing downstream inflammatory pathways, thus exerting anti-inflammatory and antidepressant effects (Tao et al., 2023). Moreover, berberine, a key bioactive compound in ZJW, has been found to regulate NLRP3 ubiquitination through Trim65, contributing to its antidepressant effects (Yang et al., 2023). These findings suggest that ZJW holds substantial promise as a treatment for depression.

Current research suggests that BA metabolism and gut microbiota are implicated in the pathological mechanisms of depression. However, it remains unclear whether ZJW exerts its antidepressant and gastrointestinal-modulating effects *via* the BA-gut microbiota axis. To explore this, the present study employed chronic social defeat stress (CSDS) to induce depression-like behaviors and gastrointestinal dysfunction in mice, aiming to investigate the potential mechanisms by which ZJW acts.

## 2 Materials and methods

### 2.1 Reagents

Zuojinwan is composed of two herbal ingredients: *Coptis chinensis* Franch (18 g, Chongqing Wanli Pharmaceutical Co., Ltd., China, Batch No.: 240101) and *Evodia rutaecarpa* (Juss.) Benth (3 g, Chongqing Wanli Pharmaceutical Co., Ltd., China, Batch No.: 240307). To prepare the active compounds, the herbs were initially soaked in water at a 1:10 ratio and heated for 60 min. A second extraction was performed with eight times the volume of water, followed by heating for 40 min. The two extracts were then combined, concentrated, and stored at 4°C. Fluoxetine hydrochloride (CAS: 56296-78-7, Sigma, United States) was used as the reference drug in the subsequent experiments. Preliminary research identified six components in ZJW using high-performance liquid chromatography (HPLC), including jatrorrhizine, coptisine, palmatine, berberine, rutaecarpine, and evodiamine (Tao et al., 2023).

### 2.2 Animal experiment

#### 2.2.1 Animals

The animal experiments were conducted in accordance with the ethical guidelines of the Animal Experiment Ethics Committee of Nanjing University of Chinese Medicine (Approval No. 202404A043). Male CD-1 mice, aged 4–6 months, were housed individually, while 7 weeks-old male C57BL/6J mice (License No. SYXK (SU) 2023-0006) were maintained under specific pathogen-free (SPF) conditions at the Experimental Animal Center of Nanjing University of Chinese Medicine. The environment was controlled at a temperature of 23 ± 3°C, humidity of 35%, and a standard light/dark cycle. Mice had *ad libitum* access to food and water.

#### 2.2.2 Experimental design

The mice were randomly assigned to one of eight experimental groups: (1) Control, (2) CSDS, (3) Fluoxetine, (4) ZJW-L, (5) ZJW-M, (6) ZJW-H, (7) Z-Gug, and (8) Z-Gug + ZJW-H. CSDS was used to induce depression-like behaviors. CD-1 mice with aggressive tendencies were chosen as aggressors. Each day, a C57BL/6J mouse was placed into a CD-1 mouse’s cage for 10 min of direct interaction, followed by a 24 h separation using a transparent divider. This process was repeated daily for 10 days, with a new CD-1 aggressor introduced each day (Li et al., 2025). Throughout the CSDS protocol, the mice were closely monitored for any signs of physical injury. No visible wounds or distress behaviors were observed, ensuring that gastrointestinal dysfunction was not caused by physical trauma.

#### 2.2.3 Group division

Following modeling, drug administration was performed. The recommended clinical dose of ZJW is 6 g/60 kg/day. Based on body surface area conversion, the equivalent doses for mice were determined: the medium dose group received 450 mg/kg, the high dose group received 910 mg/kg, and the low dose group received 225 mg/kg (Wang et al., 2023). Fluoxetine was administered at a dose of 10 mg/kg/day, and the FXR inhibitor (Z)-Guggulsterone was also administered at 10 mg/kg/day. Each group consisted of 12 mice. The control and CSDS groups received saline via oral gavage, with the gavage volume calculated as 0.1 mL/20 g of body weight. Following 3 weeks of continuous treatment, depression-like behaviors and gastrointestinal function were assessed on days 32–35. On day 36, after anesthesia was induced via intraperitoneal administration of 30 mg/kg pentobarbital sodium (Merck, United States), blood samples were collected via orbital puncture. Serum was separated by centrifugation at 12,000 × *g* for 30 min. Final euthanasia was performed by cervical dislocation in accordance with institutional ethical guidelines.

### 2.3 Behavioral tests

Video recordings were captured using Logitech Capture, and subsequent analyses were performed blindly with EthoVision XT 13. All tests followed protocols described in previous literature (Li et al., 2025).

#### 2.3.1 Social interaction test (SIT)

C57BL/6 mice were housed in an apparatus with a wire-mesh cage positioned on one side. Initially, mice were allowed to explore the open field for 2.5 min with the cage empty. In the second phase, a CD-1 mouse was placed inside the cage, and the C57BL/6 mouse was allowed 2.5 min to explore again. The time spent by the C57BL/6 mouse in the social interaction zone was recorded using tracking software. The social interaction ratio was calculated by dividing the time spent in the interaction zone during the second phase by the time spent in the same zone during the first phase.

#### 2.3.2 Sucrose preference test

Mice were administered a 2% sucrose solution for 24 h to acclimate, followed by a 24 h fasting period without access to food or water. Afterward, the animals were offered two bottles: one containing tap water and the other containing the 2% sucrose solution. After 6 h, the remaining volumes of both solutions were measured. The sucrose preference was calculated by determining the ratio of sucrose solution intake to the total volume of both sucrose solution and tap water consumed.

#### 2.3.3 Forced swimming test (FST)

Mice were placed in a cylindrical glass container filled with 15 cm of water maintained at 26°C. They were allowed to swim for 6 min, and the total duration of immobility during the last 4 min of the swim was recorded.

#### 2.3.4 Tail suspension test (TST)

Mice were suspended upside down 25 cm above the ground, with the rope positioned 1 cm from the tail. The total immobility time during the final 4 min of the procedure was recorded.

#### 2.3.5 Total gastrointestinal transit time (TGIT)

A 6% eosin solution was prepared by dissolving 0.5% sodium carboxymethyl cellulose in eosin dye. Mice were orally gavaged with 0.3 mL of the prepared eosin solution, and the time at which the first eosin-colored fecal pellet appeared was recorded as the endpoint for gastrointestinal transit assessment.

### 2.4 Gastrointestinal function tests

#### 2.4.1 Gastric residual rate

After a 24 h fasting period, mice were orally gavaged with 300 μL of black semi-solid nutritional paste. Following euthanasia, the mice were dissected, and the stomach weight (A) was recorded. The stomach contents were then removed, and the empty stomach was weighed again (B). The gastric residue rate was calculated using the following formula: Gastric residue rate (%) = (A–B)/A × 100%.

#### 2.4.2 Small intestine propulsion rate

The intestinal tissue was carefully excised, and the length of the progression of the nutritional paste was measured. The small intestine propulsion rate was calculated using the following formula: Small intestine propulsion rate (%) = (Length of nutritional paste progression/Total small intestine length) × 100%.

#### 2.4.3 Colonic motility

A 3 mm glass bead was gently inserted into the colon of the mouse, approximately 2 cm from the anus, using a glass rod. The time required for the bead to be expelled was recorded as an indicator of colonic motility. The timing started immediately after bead insertion, and the duration until bead expulsion was noted. If the bead was not expelled within 20 min, the mouse was excluded from the study (Wang et al., 2023).

### 2.5 Nissl staining

Brain tissue was fixed, paraffin-embedded, and sectioned into 20 μm thick slices. The sections were then dehydrated using an ethanol gradient, followed by staining with 1% toluidine blue for 30 min. After staining, the sections were washed, dehydrated, and cleared in xylene. Finally, the stained sections were mounted and examined under a microscope for imaging.

### 2.6 Histopathology

Intestinal tissues from mice were harvested and fixed in paraformaldehyde. The specimens were processed through a series of steps including dehydration, clearing, embedding in paraffin, sectioning into 5 μm thick slices, and deparaffinization. The sections were then stained with hematoxylin and eosin (H&E), followed by dehydration, clearing, and mounting for further microscopic examination.

### 2.7 Enzyme-linked immunosorbent assay (ELISA)

Serum samples were collected from the mice, and the following biomarkers were quantified using ELISA kits (Mlbio, China): Motilin (MTL), Gastrin (GAS), and Vasoactive Intestinal Peptide (VIP).

### 2.8 Western blot analysis

Tissue samples were homogenized and centrifuged following the protocol outlined in previous research (Tao et al., 2023). Protein concentrations were determined using a BCA assay. After denaturation, SDS-PAGE was conducted, and the proteins were transferred onto a PVDF membrane. The membrane was blocked with a rapid blocking buffer and then incubated with primary and secondary antibodies. Protein bands were detected using Enhanced Chemiluminescence (ECL) reagents. Primary antibodies included anti-GAPDH (1:5000, CST 5174) and anti-FXR (1:1000, CST 72105), with secondary antibodies being anti-mouse IgG (1:5000, CST 14709) and anti-rabbit IgG (1:5000, CST 14708).

### 2.9 Immunofluorescence staining

Tissue sections were first subjected to antigen retrieval in a 98°C water bath. The sections were then permeabilized with 10% Triton-X 100 solution. Non-specific binding was blocked by applying 5% BSA, followed by incubation. The primary antibody, anti-FXR (1:400, Abcam, ab51970), was applied and incubated overnight. The next day, the sections were incubated with the secondary antibody and stained with DAPI. After mounting, images were captured using a laser confocal microscope.

### 2.10 BA metabonomics analysis

Standard reference material was accurately weighed using an analytical balance and dissolved in water to prepare a 1 mg/mL stock solution. This stock solution was used to prepare a mixed standard solution, which was serially diluted to create a standard curve. Gradient elution was performed after setting the relevant parameters, and the samples were analyzed in multiple reaction monitoring (MRM) mode using a triple quadrupole mass spectrometer (Li X. Y. et al., 2024).

### 2.11 16S rRNA gene sequencing

DNA extraction was performed following the manufacturer’s instructions using the DNeasy PowerSoil kit (Life Technologies, Cat. No. Q32854). PCR amplification was subsequently performed, and the Ion Plus Fragment Library Kit was used for library construction. The library was then sequenced using a sequencing machine (Li et al., 2025).

### 2.12 Statistical analysis

Data are presented as mean ± standard deviation (SD). Statistical analysis was performed using GraphPad 8.0. One-Way ANOVA was applied for comparisons among multiple groups, while *t*-tests were used for two-group comparisons. A *P*-value of less than 0.05 was considered statistically significant.

## 3 Results

### 3.1 ZJW alleviates CSDS-induced depressive-like behaviors

**FIGURE 1:**
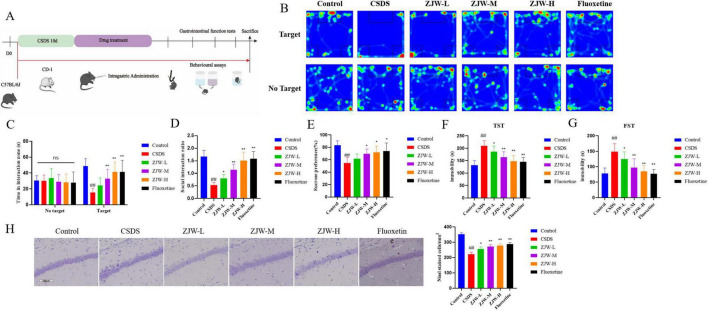
Zuojinwan (ZJW) alleviates depressive-like behavior in mice. **(A)** Experimental workflow diagram; **(B)** Social interaction test; **(C)** Time in interaction zone; **(D)** Social interaction ratio; **(E)** Sucrose preference test; **(F)** Tail suspension test; **(G)** Forced swimming test; **(H)** Nissl staining. Data are presented as mean ± SD. ##*P* < 0.01 compared to control; *P < 0.05, **P < 0.01 compared to chronic social defeat stress (CSDS).

Behavioral assessments were conducted according to the protocol outlined in Figure 1A. The results from the SIT showed a significant reduction in social interactions in the CSDS group compared to controls (*P* < 0.01). However, this deficit was notably reversed after ZJW treatment, with a marked increase in social interaction time (*P* < 0.01). In the sucrose preference test, CSDS mice displayed a reduced preference for sucrose compared to the control group (*P* < 0.01). Similarly, the TST revealed increased immobility time in the CSDS group relative to controls (*P* < 0.01). The FST also indicated longer immobility in CSDS mice compared to controls (*P* < 0.01). However, ZJW treatment reversed these depressive-like behaviors (sucrose preference test: *P* < 0.05; TST: ZJW-M and ZJW-H, *P* < 0.01; FST: ZJW-M and ZJW-H, *P* < 0.01) (Figures 1B–G). These results suggest that ZJW has the potential to mitigate depression-like symptoms induced by CSDS in mice.

Furthermore, Nissl staining was conducted to assess changes in hippocampal neurons. In the control group, hippocampal neurons appeared well-organized, with clearly defined Nissl bodies. In contrast, the CSDS group exhibited a significant reduction in Nissl-positive cells and a more disordered neuronal arrangement (*P* < 0.01). Treatment with ZJW significantly improved these changes, restoring the neuronal structure (ZJW-M and ZJW-H, *P* < 0.01) (Figure 1H). These results suggest that ZJW can reverse hippocampal neuronal damage induced by CSDS.

### 3.2 ZJW treatment of gastrointestinal dysfunction induced by depression

**FIGURE 2:**
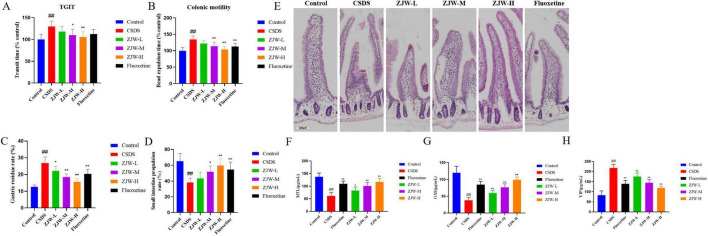
Zuojinwan (ZJW) improves gastrointestinal function in mice. **(A)** Total gastrointestinal transit time; **(B)** Colonic motility; **(C)** Gastric residue rate; **(D)** Small intestine propulsion; **(E)** H&E staining; **(F)** Motilin (MTL); **(G)** Gastrin (GAS); **(H)** Vasoactive Intestinal Peptide (VIP). Data are presented as mean ± SD. ##*P* < 0.01 compared to control; *P < 0.05, **P < 0.01 compared to chronic social defeat stress (CSDS).

Given that CSDS-induced depression is frequently associated with gastrointestinal dysfunction, gastrointestinal motility was further assessed. The TGIT was significantly prolonged in the CSDS group compared to controls (*P* < 0.01). However, ZJW treatment reduced the transit time in a dose-dependent manner (ZJW-M, *P* < 0.05; ZJW-H, *P* < 0.01). Additional assessments of colonic motility, gastric residue, and small intestine propulsion showed that CSDS mice exhibited slowed colonic motility (*P* < 0.01), increased gastric residue (*P* < 0.01), and reduced small intestine propulsion compared to controls (*P* < 0.01). ZJW treatment improved these gastrointestinal impairments (Figures 2A–D) (TGIT: ZJW-M, *P* < 0.05; ZJW-H, *P* < 0.01; colonic motility: *P* < 0.01; gastric residue: ZJW-M and ZJW-H, *P* < 0.01; small intestine propulsion: ZJW-M, *P* < 0.05; ZJW-H, *P* < 0.01). These results suggest that ZJW can alleviate gastrointestinal dysfunction associated with CSDS-induced depression.

Histological examination using H&E staining revealed pathological changes in intestinal tissue. In the CSDS group, the intestinal villi were markedly shortened. However, after ZJW treatment, the epithelial cell arrangement became more organized, and the villous structure showed significant improvement (Figure 2E). These results indicate that ZJW can mitigate the pathological alterations in intestinal tissue induced by CSDS.

Given the gastrointestinal dysfunction accompanied by hormone imbalances, ELISA was employed to measure gastrointestinal hormones, including MTL, GAS, and VIP. The results showed that, compared to the control group, the CSDS group had significantly decreased MTL (*P* < 0.01) and GAS (*P* < 0.01) levels, along with elevated VIP levels (*P* < 0.01). After ZJW treatment, both MTL (ZJW-M and ZJW-H, *P* < 0.01) and GAS (ZJW-M and ZJW-H, *P* < 0.01) levels were significantly increased, while VIP levels (ZJW-M and ZJW-H, *P* < 0.01) were markedly reduced (Figures 2F–H).

### 3.3 ZJW improves depression and gastrointestinal dysfunction by regulating FXR expression

**FIGURE 3:**
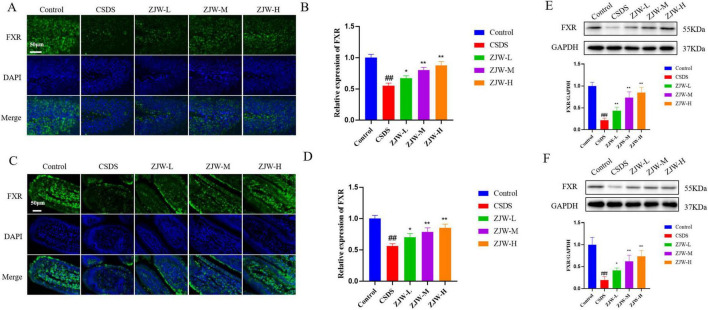
Effects of Zuojinwan (ZJW) on farnesoid X receptor (FXR) protein expression. **(A,B)** Hippocampal immunofluorescence; **(C,D)** Intestinal immunofluorescence; **(E)** Hippocampal Western Blot; **(F)** Intestinal Western Blot. Data are presented as mean ± SD. ##*P* < 0.01 compared to control; *P < 0.05, **P < 0.01 compared to chronic social defeat stress (CSDS).

Immunofluorescence was employed to evaluate the expression of FXR in both the intestine and hippocampus. In the hippocampus, FXR expression was significantly reduced in the CSDS group compared to the control group (Figures 3A, B) (*P* < 0.01). A similar decrease in FXR expression was observed in the intestine of the CSDS group (Figures 3C, D) (*P* < 0.01). However, treatment with ZJW resulted in a notable increase in FXR expression in both the hippocampus and intestine (ZJW-M and ZJW-H, *P* < 0.01). To further validate these results, Western blot analysis was performed, yielding consistent findings with the immunofluorescence data (Figures 3E, F) (ZJW-M and ZJW-H, *P* < 0.01). These results suggest that ZJW upregulates FXR expression in both the hippocampus and intestine, which may contribute to its therapeutic effects.

### 3.4 ZJW treatment alters bile acid metabolism

**FIGURE 4:**
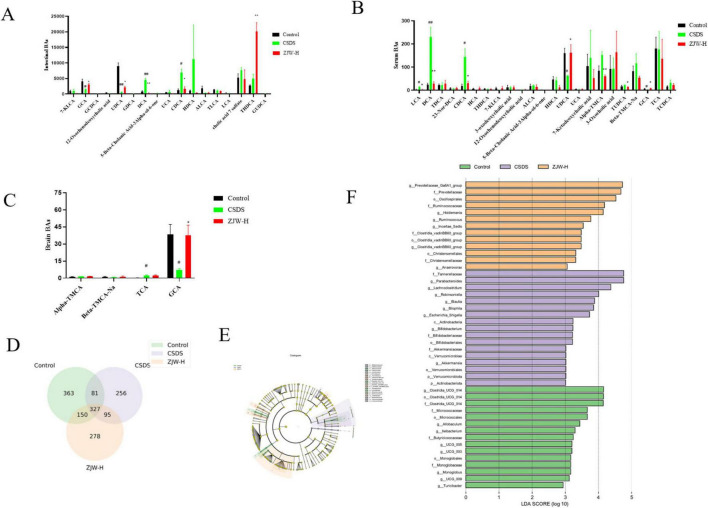
Effects of Zuojinwan (ZJW) on bile acids and gut microbiota. **(A)** Intestinal bile acids; **(B)** Serum bile acids; **(C)** Hippocampal bile acids; **(D)** Venn diagram; **(E)** Differential species plot; **(F)** Differential species score plot. Data are presented as mean ± SD. #*P* < 0.05, ##*P* < 0.01 compared to control; *P < 0.05, **P < 0.01 compared to chronic social defeat stress (CSDS).

Given that FXR regulates BA synthesis, BA metabolism in the serum, hippocampus, and gut was assessed. BA levels were quantified using UPLC-MS/MS analysis. Significant differences in BA composition were observed between the CSDS and control groups. Notably, Glycocholic acid (GCA) levels in the hippocampus were significantly altered (*P* < 0.05). Additionally, levels of GCA, deoxycholic acid (DCA) (*P* < 0.01), ursodeoxycholic acid (UDCA) (*P* < 0.05), and chenodeoxycholic acid (CDCA) (*P* < 0.05) in the gut and serum also showed significant variations (*P* < 0.05). After ZJW treatment, these BA levels in the serum, gut, and hippocampus were reversed (Figures 4A–C) (GCA, *P* < 0.05; DCA, *P* < 0.01; UDCA, *P* < 0.05; CDCA, *P* < 0.05). These results indicate that ZJW helps restore BA homeostasis by modulating BA metabolism.

## 3.5 ZJW treatment alters the gut microbiota

### 3.5.1 Inter-group species differences

Venn diagram analysis was conducted to identify unique and shared operational taxonomic units (OTUs) across the groups. A total of 327 OTUs were common to all groups. The Control, Model, and ZJW groups showed 363, 256, and 278 unique OTUs, respectively (Figure 4D). These results underscore the distinct microbial profiles associated with each group and suggest that ZJW may influence gut microbiota composition.

To further explore microbial differences, Linear Discriminant Analysis Effect Size (LEfSe) was used to identify differentially abundant taxa between the groups. The LDA score represents the strength of each microorganism’s contribution to observed differences, with higher scores indicating a greater impact. LEfSe analysis identified 45 biomarker taxa with an LDA score greater than three. Among the groups, the Control, Model, and ZJW groups exhibited 13, 17, and 15 dominant bacterial taxa, respectively. In the Model group, the predominant taxa included *Tannerellaceae*, *Parabacteroides*, *Lachnoclostridium*, *Robinsoniella*, and *Bilophila*. In contrast, the ZJW group displayed a higher abundance of *Prevotellaceae*, *Oscillospirales*, *Ruminococcaceae*, and *Holdemania* (Figures 4E, F). These results suggest that ZJW may promote a more favorable gut microbiota composition.

#### 3.5.2 Alpha and beta diversity analysis

**FIGURE 5:**
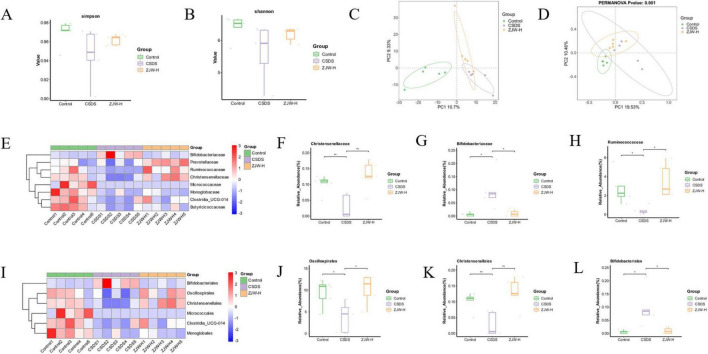
Effects of Zuojinwan (ZJW) on the gut microbiota. **(A)** Simpson index; **(B)** Shannon index; **(C)** PCA analysis; **(D)** Principal coordinates analysis (PCoA) analysis; **(E)** Family-level differential species heatmap; **(F–H)** Family-level differential species boxplot; **(I)** Order-level differential species heatmap; **(J–L)** Order-level differential species boxplot. Data are presented as mean ± SD. *P < 0.05, **P < 0.01.

To evaluate the richness and diversity of microbial communities, the Shannon and Simpson indices were calculated. Compared to the control group, the CSDS group exhibited reduced community richness and diversity. However, after ZJW treatment, both the richness and diversity of the community were significantly improved relative to the CSDS group (Figures 5A, B). Additionally, principal component analysis (PCA) and principal coordinates analysis (PCoA) were performed to assess beta diversity. The PCA plot revealed that the microbial composition of each group was relatively similar. In contrast, the PCoA results showed a clear separation between the groups, with statistical significance (*P* < 0.05) (Figures 5C, D). These results indicate that ZJW treatment enhances microbial community diversity and helps restore the microbial balance disrupted by CSDS.

#### 3.5.3 Impact of ZJW on the microbial composition at the family and order levels

Microbial composition was further assessed at the order and family levels. At the order level, ZJW treatment led to an increase in the abundance of *Oscillospirales* and *Christensenellales*, while the abundance of *Bifidobacteriales* decreased compared to the CSDS group. At the family level, ZJW treatment resulted in an increase in the abundance of *Ruminococcaceae* and *Christensenellaceae*, while *Bifidobacteriaceae* abundance was reduced (Figures 5E–L). These results suggest that ZJW may regulate gut health by modulating the abundance of these six bacterial taxa, increasing beneficial bacteria while decreasing harmful ones.

### 3.6 Inhibition of FXR activation abolished the protective effects of ZJW against depression and gastrointestinal dysfunction in mice

**FIGURE 6:**
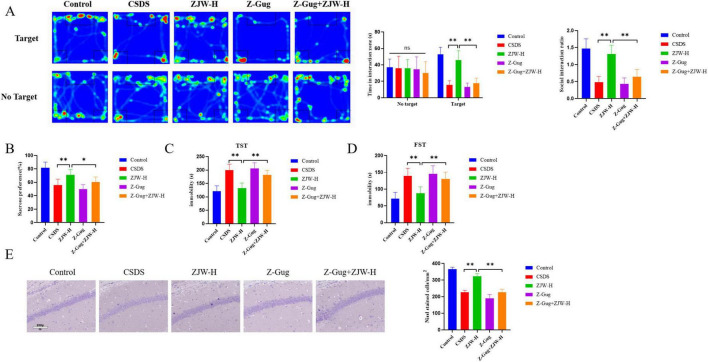
Inhibition of farnesoid X receptor (FXR) activation abolished the protective effects of Zuojinwan (ZJW) against depression in mice. **(A)** Social interaction test; **(B)** Sucrose preference test; **(C)** Tail suspension test; **(D)** Forced swimming Test; **(E)** Nissl staining. Data are presented as mean ± SD. *P < 0.05, **P < 0.01.

**FIGURE 7:**
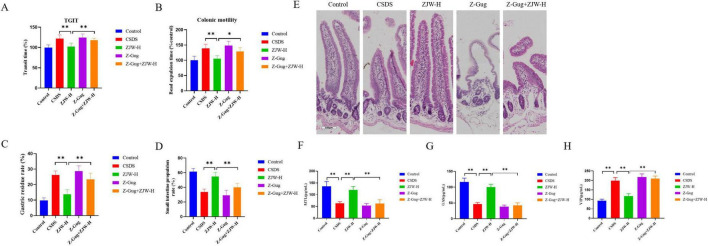
Inhibition of farnesoid X receptor (FXR) activation abolished the protective effects of Zuojinwan (ZJW) against gastrointestinal dysfunction in mice. **(A)** Total gastrointestinal transit time; **(B)** Colonic motility; **(C)** Gastric residue rate; **(D)** Small intestine propulsion; **(E)** H&E staining; **(F)** Motilin (MTL); **(G)** Gastrin (GAS); **(H)** Vasoactive Intestinal Peptide (VIP). Data are presented as mean ± SD. *P < 0.05, **P < 0.01.

In this study, the FXR inhibitor was co-administered with ZJW to assess their combined effects on depressive-like behaviors and gastrointestinal dysfunction in mice. Co-treatment with the FXR inhibitor attenuated ZJW’s antidepressant effects compared to the ZJW monotherapy group. This was evidenced by reduced social interaction time (*P* < 0.01) and sucrose consumption (*P* < 0.01), prolonged immobility duration in the TST (*P* < 0.01) and FST (*P* < 0.01), and limited recovery of hippocampal neuronal injury (*P* < 0.01) (Figures 6A–E). Furthermore, co-administration of the FXR inhibitor also impaired ZJW’s therapeutic efficacy against gastrointestinal dysfunction, as demonstrated by elevated TGIT time (*P* < 0.01), prolonged bead expulsion time (*P* < 0.05), increased gastric residue levels (*P* < 0.01), reduced small intestine propulsion rate (*P* < 0.01), and incomplete resolution of intestinal tissue injury (Figures 7A–E). ELISA results showed that compared to ZJW alone, the co-administration of the FXR inhibitor with ZJW led to decreased MTL (*P* < 0.01) and GAS levels (*P* < 0.01), along with elevated VIP levels (*P* < 0.01), thereby compromising ZJW’s therapeutic effects (Figures 7F–H).

## 4 Discussion

The global incidence of depression has been steadily increasing, becoming a significant health concern worldwide (Friedrich, 2017). While the exact mechanisms underlying its development remain incompletely understood, research suggests that BAs and the gut microbiota may play pivotal roles in the onset of depression (Sun et al., 2022). Given the established correlation between depression and gastrointestinal dysfunction, this study explored the pathogenesis of depression accompanied by gastrointestinal disorders (Haj et al., 2018). However, the mechanisms by which FXR modulates depression and gastrointestinal dysfunction *via* BA-gut microbiota interactions remain unclear. Our findings indicate that FXR influences disease progression through the regulation of BA-gut microbiota interactions. Moreover, the TCM formula ZJW enhances FXR expression, thereby modulating BA-gut microbiota interactions to treat depressive-like behaviors and gastrointestinal dysfunction in mice. This discovery unveils a natural therapeutic approach for treating depression accompanied by gastrointestinal disorders.

Depressive-like behaviors in mice are often associated with hippocampal neuronal damage, while gastrointestinal dysfunction correlates with intestinal tissue injury (Tao et al., 2023; Wang et al., 2024). Our findings corroborate these observations, as model mice exhibited hippocampal neuronal damage alongside gastrointestinal dysfunction and concurrent intestinal tissue injury. FXR has been shown to be expressed in a tissue-specific manner in both the liver and intestine, where it plays a vital role in regulating BA synthesis and maintaining BA balance (Stofan and Guo, 2020). Additionally, FXR is involved in multiple biological processes, including cell growth, metabolism, and apoptosis (Chawla et al., 2001). Recent studies suggest that FXR may be implicated in the development of depression. Specifically, in CSDS-induced depressed mice, FXR expression is diminished in the prefrontal cortex, and FXR knockout mice exhibit behaviors characteristic of depression (Bao et al., 2021). Other studies have also reported a reduction in FXR protein levels in the ileum of depressed mice (Wang et al., 2024). Furthermore, activating FXR expression has been shown to exert antidepressant effects (Li C. et al., 2024). These findings are consistent with our results, where a decrease in FXR protein levels was observed in both brain and intestinal tissues of depressed mice. Moreover, after the administration of an FXR antagonist, depressive-like behavior in the mice worsened. FXR is closely related to intestinal epithelial barrier integrity and inflammatory responses. The FXR agonist, Nelumal A, has been shown to effectively improve tight junctions in the intestine and restore epithelial barrier function (Miyazaki et al., 2021). Our study also revealed gastrointestinal dysfunction in depressed mice, accompanied by reduced FXR protein expression, with further exacerbation of gastrointestinal dysfunction following FXR antagonist treatment. Based on these observations, it is hypothesized that FXR may serve as a potential target for treating depression.

Given FXR’s pivotal role in regulating both the synthesis and composition of BAs, alterations in BA levels were further examined in mice. BAs are primarily synthesized in the liver, secreted into the small intestine *via* the bile ducts, and subsequently reabsorbed in the ileum and colon before being transported back to the liver. Thus, BA concentrations in peripheral blood are typically low (Fiorucci et al., 2024). Recent studies, however, have identified BAs in the brain (Varma et al., 2021), indicating their potential to either cross the blood-brain barrier or be synthesized directly within the central nervous system (Mano et al., 2004). Moreover, research demonstrates that BAs can influence both brain and gastrointestinal functions under various physiological and pathological conditions (Wu et al., 2024). These findings highlight the potential role of BAs in modulating central nervous system and gut health, possibly *via* FXR-mediated pathways.

This study identified the presence of BAs in the brain, gut, and serum, with significant alterations in their composition and levels observed in depressed mice. In line with our findings, other studies have reported notable changes in BA composition in the serum and hypothalamus of depression-susceptible mice compared to control groups. Clinical evidence further supports these observations, showing altered BA levels in the serum of patients with depression (Li X. Y. et al., 2024). Similarly, BAs are closely associated with functional dyspepsia (Wauters et al., 2021). Our results demonstrated an increase in hydrophobic BAs, accompanied by a reduction in beneficial BAs such as GCA and UDCA. This is consistent with other studies indicating that hydrophobic BAs contribute to gastrointestinal dysfunction (Ruiz-Malagon et al., 2022). Furthermore, Qu et al. (2022) reported significantly elevated levels of hydrophobic BAs in depressed mice. These findings suggest that BA homeostasis disruption may play a role in depressive-like behaviors and gastrointestinal dysfunction. Our study further revealed that depressed mice exhibit gastrointestinal dysfunction alongside altered BA composition and levels. Therefore, FXR may be crucial in regulating BAs to address depression associated with gastrointestinal dysfunction.

BAs influence the diversity and richness of the gut microbiota (Guo et al., 2022). To further explore this, changes in the gut microbiota of mice were examined. The gut microbiota has been implicated in the development of mental health disorders, including depression, through mechanisms such as neurodevelopment (Du Toit, 2022; Khaledi et al., 2024; Sun et al., 2023). Clinical studies have shown significant differences in the gut microbiota between individuals with major depressive disorder and healthy controls (Fontana et al., 2020). Similarly, alterations in the gut microbiota have been observed in animal models of depression, with specific microbial species potentially contributing to depressive behaviors (Fontana et al., 2020). Furthermore, significant shifts in the gut microbiota have been documented in rats with gastrointestinal dysfunction, including conditions such as functional dyspepsia (Bai et al., 2023).

In this study, notable changes were observed in the gut microbiota of depressed mice, particularly at the genus and family levels. Three microbial groups—*Oscillospirales*, *Christensenellales*, and *Bifidobacteriales*—underwent significant alterations. Additionally, 16S sequencing revealed increased Oscillospirales (anti-inflammatory) and decreased *Parabacteroides* (stress-responsive) in ZJW-treated mice. These findings suggest that the gut microbiota plays a critical role in the development of both depression and gastrointestinal disorders, with a strong association with the aforementioned microbial groups. *Oscillospira* is generally considered beneficial for microbiota stability and host health (Xiang et al., 2024). *Christensenellales* has potential probiotic functions, including anti-inflammatory effects (Huang et al., 2024). Although *Bifidobacteriales* is typically beneficial, it was significantly increased in the model group, likely due to the heightened stress response, which prompts the production of more *Bifidobacteriales* to mitigate the harmful environment. *Parabacteroides*, a genus within the Bacteroidetes phylum, has been shown to induce depressive-like behaviors in preclinical studies, with *P. distasonis* specifically implicated (Gomez-Nguyen et al., 2021). Our results demonstrated a significant increase in Parabacteroides abundance in the model group, which was notably reduced following ZJW administration. The decreased abundance of beneficial bacteria such as *Oscillospirales*, *Parabacteroides*, and *Christensenellales* may contribute to disease development.

Zuojinwan, a traditional natural medicine, is widely utilized in clinical practice. As a dual-ingredient TCM formula, ZJW may act synergistically through multiple pathways: (1) FXR activation; (2) gut microbiota modulation; and (3) anti-inflammatory effects (Tao et al., 2023). This multi-target mechanism distinguishes ZJW from single-compound antidepressants. Our study found that ZJW improves depressive-like behaviors and gastrointestinal dysfunction in mice. Hippocampal Nissl staining revealed ZJW-mediated restoration of neuronal integrity, correlating with improvements in depressive-like behaviors. Similarly, intestinal H&E staining showed ZJW-reversed intestinal injury, paralleling enhanced gastrointestinal function. Furthermore, ZJW promotes FXR protein expression, alters BA composition and content, and influences the gut microbiota. 16S sequencing identified increased *Oscillospirales* (anti-inflammatory) and decreased *Parabacteroides* (stress-responsive) in ZJW-treated mice. To further investigate the mechanism, an FXR antagonist was co-administered with ZJW. Results showed that ZJW significantly improved depressive-like behaviors and gastrointestinal dysfunction even in the presence of the FXR antagonist. The co-administration of an FXR inhibitor with ZJW attenuated its therapeutic efficacy in alleviating depression and gastrointestinal dysfunction. These findings suggest that ZJW may regulate depressive-like behaviors and gastrointestinal dysfunction through the FXR-BA-gut microbiota axis.

However, some limitations exist in this study. While differential BAs and microbial groups were identified, further research is needed to fully elucidate the underlying mechanisms. Future studies should explore these findings in greater depth. Nonetheless, our preliminary research indicates that the liver-brain-gut axis may serve as a potential therapeutic target for related diseases, providing a foundation for future research in this area.

## Data Availability

The raw data supporting the conclusions of this article will be made available by the authors, without undue reservation.
